# Is the effect of a high-flow nasal cannula after extubation evaluated by electrical impedance tomography applicable to clinical practice?

**DOI:** 10.1186/s13054-020-02899-2

**Published:** 2020-04-28

**Authors:** Wataru Matsuda

**Affiliations:** Department of Emergency Medicine and Critical Care, Center Hospital of the National Center for Global Health and Medicine, 1-21-1 Toyama, Shinjuku, Tokyo, 162-8655 Japan

To the Editor,

The recently published article by Zhang et al. [1] in *Critical Care* reported that electrical impedance tomography (EIT) may have the potential to identify post-extubation patients for whom high-flow nasal cannula (HFNC) therapy is beneficial. While I applaud their work, I would like to discuss several issues regarding the application of this approach in clinical settings, particularly the treatment protocol on which their measurements were based.

First, I would like to consider the flow rate of HFNC therapy. The authors used conventional oxygen therapy as a baseline and subsequently adjusted the HFNC flow rate at 20, 40, and 60 L/min increments. However, the flow rate of oxygen therapy should be set high to prevent inhalant gas from being diluted by air [2]. Additionally, the higher the HFNC flow rate, the lower the work of breathing [3]. Consequently, I believe it is appropriate to set a high flow rate soon after extubation and then gradually reduce it to about 30 L/min. A recently published large randomized trial compared non-invasive ventilation with HFNC vs HFNC alone using a similar protocol [4]. Furthermore, some physicians have attempted to place the patient on HFNC therapy before extubation to prevent alveolar collapse post-extubation. The authors’ protocol thus seems to differ from actual practice.

Second, this was a single-arm study. The change in end-expiratory lung impedance increased with each increase in the HFNC flow rate. However, after extubation, the tidal volume may increase spontaneously because of recovery from sedation or reduction of dead space from the intubation tube. This change may thus occur with conventional oxygen therapy as well. Therefore, to clearly demonstrate the benefit of increased flow rate, it is preferable to compare HFNC with conventional oxygen therapy.

Third, lung overdistention was observed in some subjects. The authors found that EIT could detect the risk of lung overdistention from HFNC. Measurements were obtained in the semi-recumbent position, and lung overdistention was observed in mainly the ventral region. Therefore, this risk could be avoided by using the prone position. In patients with acute respiratory distress syndrome, the prone position is thought to help improve lung heterogeneity and reduce regional overdistention. Also, a small study showed that the combination of HFNC and prone ventilation was more favorable than HFNC alone [5]. Therefore, I believe that EIT can be useful for exploring optimal positioning during HFNC therapy.

## Authors’ response

Huaiwu He, Yun Long

We read the letter from Wataru Matsuda with interest and are delighted to respond to their questions and comments.

First, our study compared different flow rates in an effort to assess an optimal flow rate (induce recruitment with minimal overdistension). Hence, a small step of incremental flow rate (20 L/min) was selected. Moreover, an initial high flow might bring uncomfortable to patients who were at relatively normal respiratory status without obvious hypoxia. Furthermore, the increasing flow rate process had been reported in the previous study of EIT [6].

Second, we agreed that the investigation of the difference of HFNC and conventional oxygen therapy was meaningful. The baseline could be taken as conventional oxygen therapy in our study. Actually, the recruited pixel assessed by EIT was compared to the baseline. Therefore, the result could effectively reflect the lung response to HENC. Using EIT to guide the mechanical ventilator setting has become popular in clinical practice [7].

Third, lung overdistension is an interesting topic. A recent clinical case reported HENC could cause pulmonary hyperinflation with mild pneumomediastinum assessed by high-resolution chest computed tomography scan [8] in a patient with bronchiolitis obliterans syndrome. Moreover, Kotani et al. reported the regional overdistension was detected at a prone position in an ARDS patient with a low tidal volume [9]. We proposed a novel EIT method to assess lung overdistension in the spontaneous breath condition [1]. Further studies are required to using EIT to assess the effect of HENC on lung overdistension at the prone position.

## Data Availability

Not applicable
